# Distribution of the subtendons in the midportion of the Achilles tendon revealed in vivo on MRI

**DOI:** 10.1038/s41598-020-73345-0

**Published:** 2020-10-01

**Authors:** Paweł Szaro, Walter Cifuentes Ramirez, Simon Borkmann, Alexander Bengtsson, Mateusz Polaczek, Bogdan Ciszek

**Affiliations:** 1 Department of Radiology, Institute of Clinical Sciences, Sahlgrenska Academy, University of Gothenburg, Göteborgsvägen 31, 431 80 Gothenburg, Sweden; 2 Department of Musculoskeletal Radiology, Sahlgrenska University Hospital, Gothenburg, Sweden; 3 Third Department of Lung Diseases and Oncology, National Tuberculosis and Lung Diseases Research Institute, Medical University of Warsaw, Warsaw, Poland; 4 Department of Descriptive and Clinical Anatomy, Medical University of Warsaw, Warsaw, Poland

**Keywords:** Medical research, Trauma, Anatomy, Muscle, Tendons

## Abstract

The aim of the study was to check if the subtendons of the Achilles tendon can be identified in vivo on MRI in the midportion of the tendon. The relation of the plantaris tendon to the Achilles tendon was also examined. A retrospective study of 200 MRI of ankle joints including the Achilles tendon was conducted. Statistical analysis of the correlation between the possibility of identifying the subtendons and the side, gender, presence of the central soleus tendon and plantaris tendon variation was performed. The inter-observer agreement between two reviewers in their evaluation of the subtendons was assessed using kappa statistics. The subtendon from the lateral head of the gastrocnemius muscle was identified in 65% (k = 0.63) and was located in the anterior part of the Achilles tendon. The subtendon from the soleus muscle was recognized in 12% (k = 0.75) comprising anterior part of the tendon. In 6% the subtendon from the medial head of the gastrocnemius muscle was identified (k = 0.58). The central soleus tendon was identified in 85% of cases. Statistical analysis shows the weak correlation of the presence of the central soleus tendon and the possibility of identifying the subtendon from the soleus muscle. The plantaris tendon was directly related to the insertion of the Achilles tendon in 42.5%. Identification of the subtendons of the Achilles tendon on MRI is challenging, and most often it is only possible to find the subtendon of the lateral head of the gastrocnemius muscle.

## Introduction

The Achilles tendon is composed of twisted subtendons corresponding to each part of the triceps surae muscle. The internal structure of the muscle and tendon has been studied intensively over the last few years because the incidence of injuries has increased almost three times over the past 30 years (18 vs 55 per 100,000)^1–3^. The fascicular structure of the Achilles tendon is well known and documented on cadavers^4^, however, there is no corresponding radiological work on large material in magnetic resonance imaging (MRI). The Achilles tendon subtendons initially run vertically, corresponding to the position of the muscle bellies. In the midportion of the tendon, a clear rotation is observed so that subtendon from the lateral head of the gastrocnemius muscle (S-LGC) is found in the anterior part while subtendon from the medial head (S-MGC) in the posterior part of the tendon^4,5^. The subtendon from the soleus muscle (S-Sol), which is a prolongation of the soleus central tendon (T-Sol)^6–9^ is located in the medial and anterior part of the tendon. Subtendons are separated by thin membranes which makes it possible to separate them during dissection^10^.

The midportion of the Achilles tendon is located about 2–7 cm above the calcaneal tuber and is a typical site for tendinopathy and injury. In most cases, no diagnostic imaging is needed because the clinical examination is sufficient^11^. However, diagnostic imaging supports the diagnosis of Achilles tendinopathy, which is the most common cause of pain of the Achilles tendon in athletes and the general population^8,12^. MRI is a very sensitive method for detecting pathological changes in the Achilles tendon because pathological changes can easily be identified in the tendon showing a low signal on T2-weighted and proton density sequences^10,13^. The use of ultra-short pulse sequences techniques allows an increase in the sensitivity of the detecting pathologies in the enthesis organ^14^. Ultrasound is a routinely used imaging method of the pathology of the Achilles tendon^8^, but due to the fact that our work applies to the use of MRI, we will not discuss ultrasound more broadly.

The aim of the study was to evaluate the possibility of identifying subtendons in the midportion of the Achilles tendon in vivo on MRI, assessing the rotation of the subtendons and the relation of the plantaris tendon to the Achilles tendon.

## Results

### Identification of the subtendons

**Figure 1:**
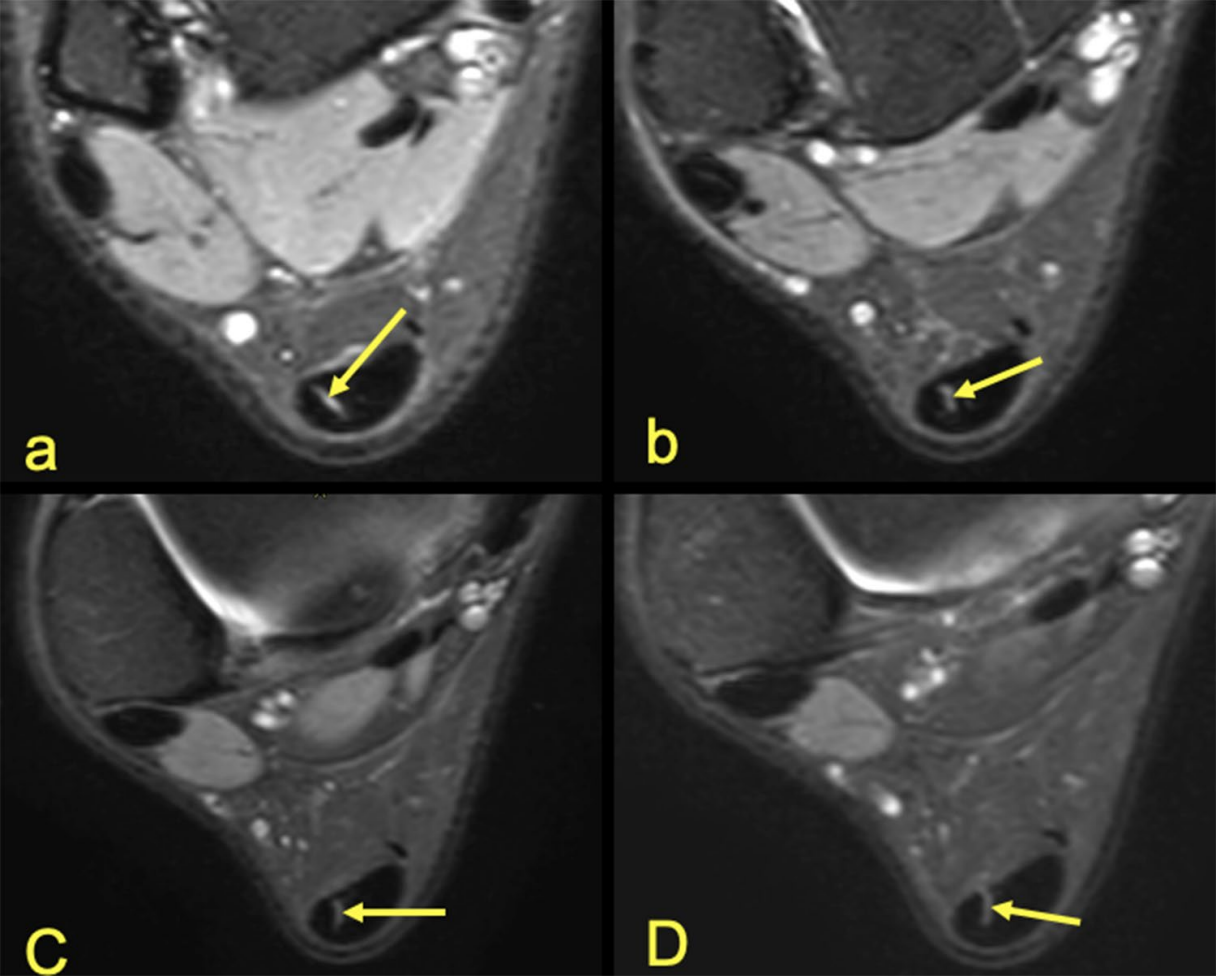
MRI right ankle, PD FS at the level of the talus. (**a**)—The highest section, (**d**)—the lowest section. A 42-year-old male with ankle sprain without trauma. The arrow marks a narrow septation between the subtendon from the lateral head of the gastrocnemius muscle and the other subtendons of the Achilles tendon.

**Figure 2:**
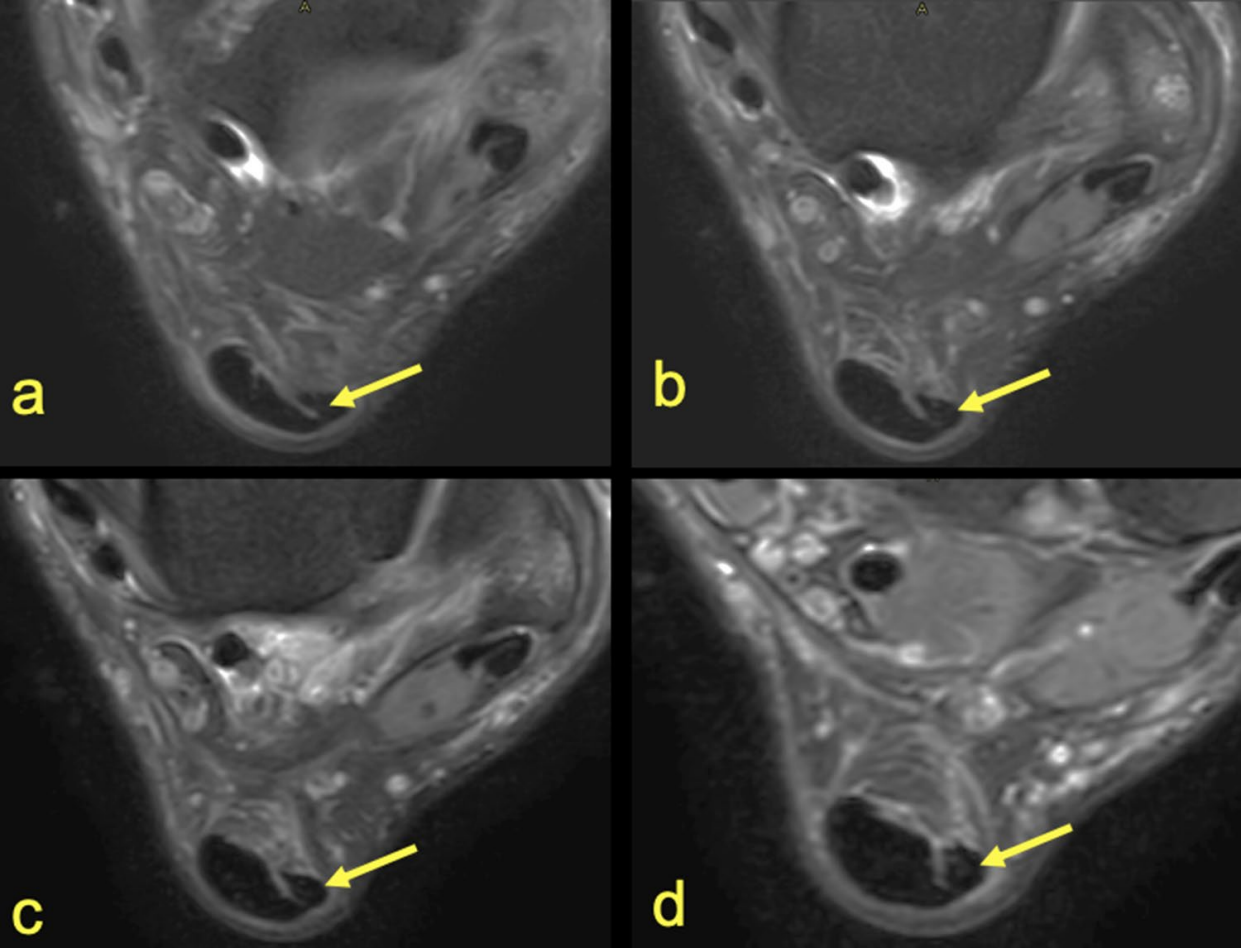
MRI left ankle, PD FS at the level of the talus (**a**)—the highest section, (**d**)—the lowest section. A 35-year-old male 2 weeks after ankle sprain, pain laterally. The arrow marks the S-LGC.

Identification of the separation between the subtendons of the Achilles tendon was possible in n = 141 cases (70.5%) and occurred on the anterior part of the tendon (Table 1). Identification of the subtendons was possible due to the presence of thin high-signal planes separating the subtendons, which usually occur around the S-LGC (Figs. 1, 2, 4). The highest agreement between reviewers was in the case of the S-Sol (Table 2) while the lowest was seen in identification of the S-MGC. Final determination of the subtendons was made by consensus (Table 1).

**Table 1:** Results of the ability to distinguish the subtendons of the Achilles tendon and the plantaris connection to the Achilles tendon. Side (R—right, L—left).

Sex	Side	Subtendon of the Achilles tendon			T-Sol	The plantaris tendon					
		S-MGC	S-LGC	S-Sol							
						Absence	Type 1	Type 2	Type 3	Type 4	Type 5
Females	R	2	33	8	48	6	0	2	18	19	4
	L	3	39	6	41	5	2	4	17	16	4
Males	R	4	30	7	43	5	1	3	20	23	5
	L	3	28	3	38	4	1	3	22	15	1
n		12	130	24	170	20	4	12	77	73	14
%		6%	65%	12%	85%	10%	2%	6%	38.5%	36.5%	7%

The type of plantaris tendon is according to Olewnik et al.^15^.

**Table 2:** Inter- and intra-observer reliability of identification of subtendons of the Achilles tendon and variations of the plantaris tendon.

	S-MGC	S-LGC	S-Sol	T-Sol	The plantaris tendon					
					Absent	Type 1	Type 2	Type 3	Type 4	Type 5
Intraobserver	0.86	0.86	0.92	0.85	0.93	0.81	0.99	0.90	0.93	0.92
Interobserver	0.58	0.63	0.75	0.68	0.71	0.53	0.97	0.72	0.71	0.56

**Figure 3:**
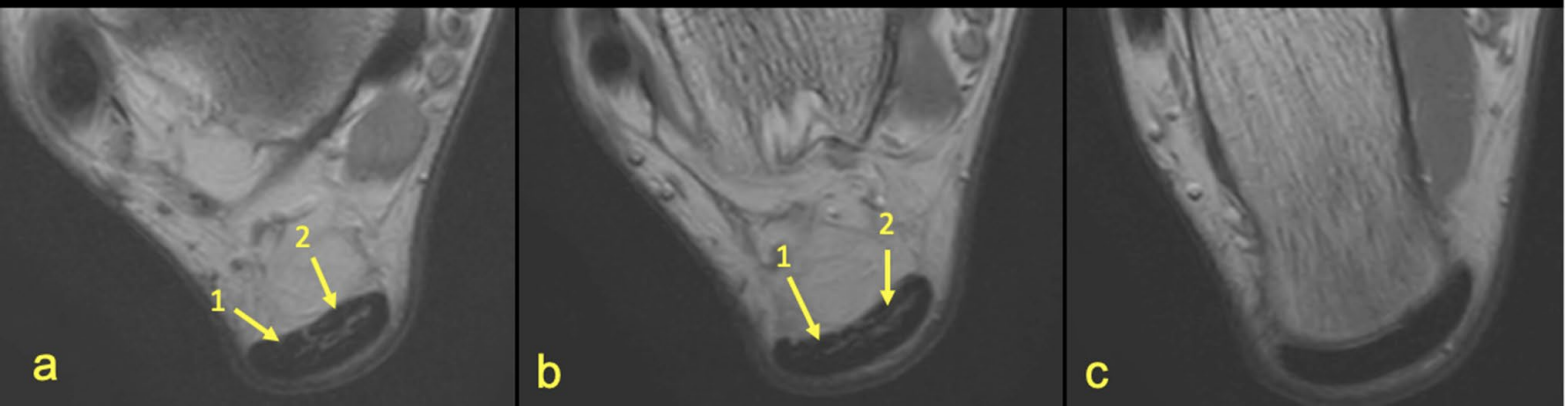
MRI right ankle, PD FS at the level of the talus (**a**), superiorly to the calcaneus (**b**) and the Achilles tendon insertion (**c**). A 23-year-old male with a suspected anterior talofibular ligament rupture. 1—S-LGC, 2—S-Sol. It is not possible to clearly distinguish S-MGC.

**Figure 4:**
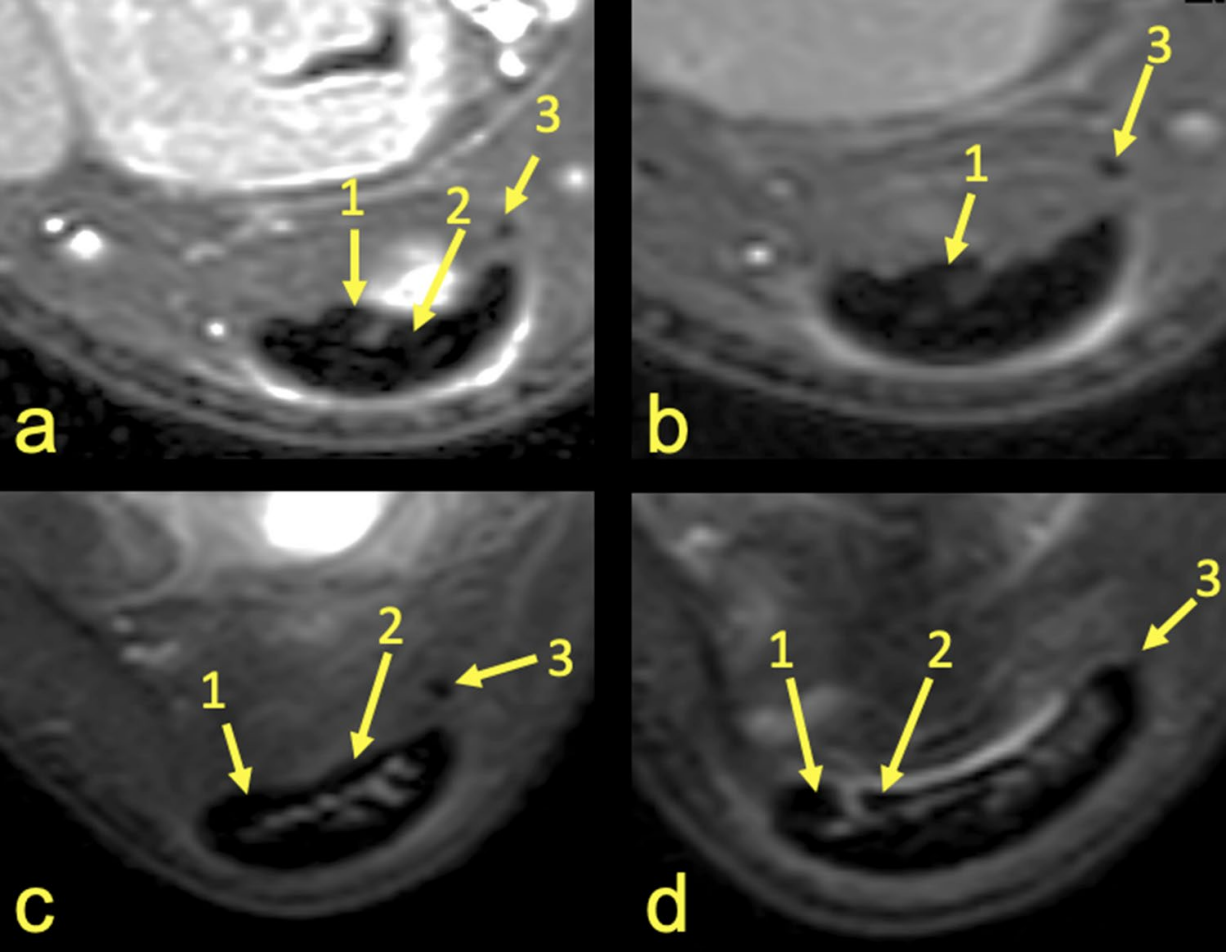
MRI right ankle, PD FS at the level of the talus (**a**–**c**), (**a**)—the highest section, (**d**)—the lowest section and at the level of the deep calcaneal bursa just above the calcaneus (**d**). A 17-year-old female 1 week after ankle sprain, pain posteriorly to the lateral malleolus. 1—S-Sol, 2—S-LGC, 3—the plantaris tendon (type 4).

The subtendons from the S-LGC was clearly identified in n = 130 cases (Figs. 1, 2, 3). The S-Sol was recognized in n = 24 cases, while the S-MGC in n = 12 cases (Fig. 4). Rarely could only the S-Sol (n = 5) or S-MGC (n = 1) be identified.

Identification of the subtendons of the Achilles tendon was possible in the midportion of the tendon (Figs. 1, 2, 3, 4, 5, 6, 7, 8) whereas septation between the subtendons was not visible in the insertion in any of the cases (Figs. 7D, 8F).

**Figure 5:**
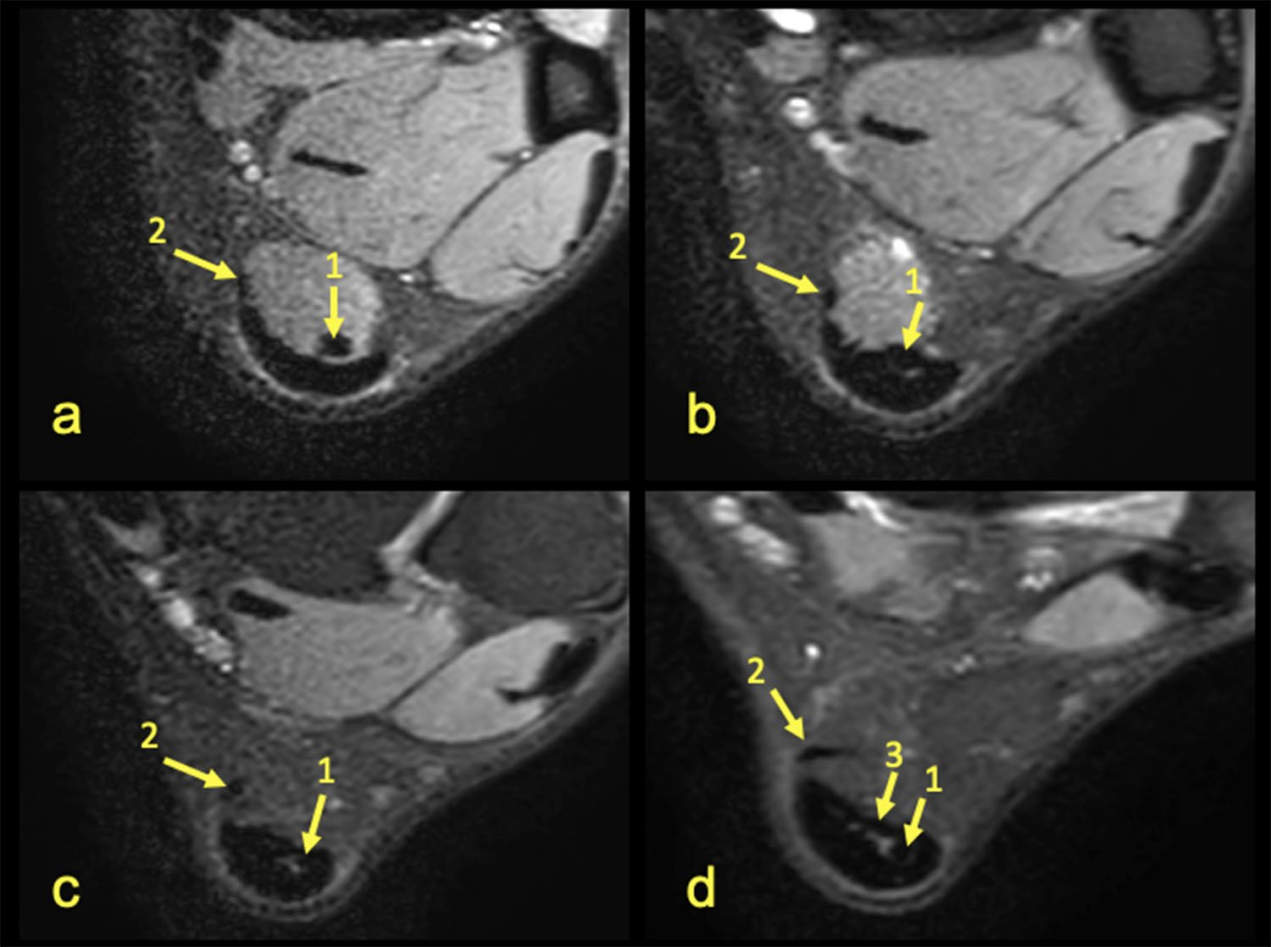
MRI left ankle, PD FS at the level of the talus; (**a**)—the highest section, (**d**)—the lowest section. A 48-year-old male presented with the anterior ankle pain. 1—S-Sol, 2—the plantaris tendon (type 5), 3—S-LGC.

**Figure 6:**
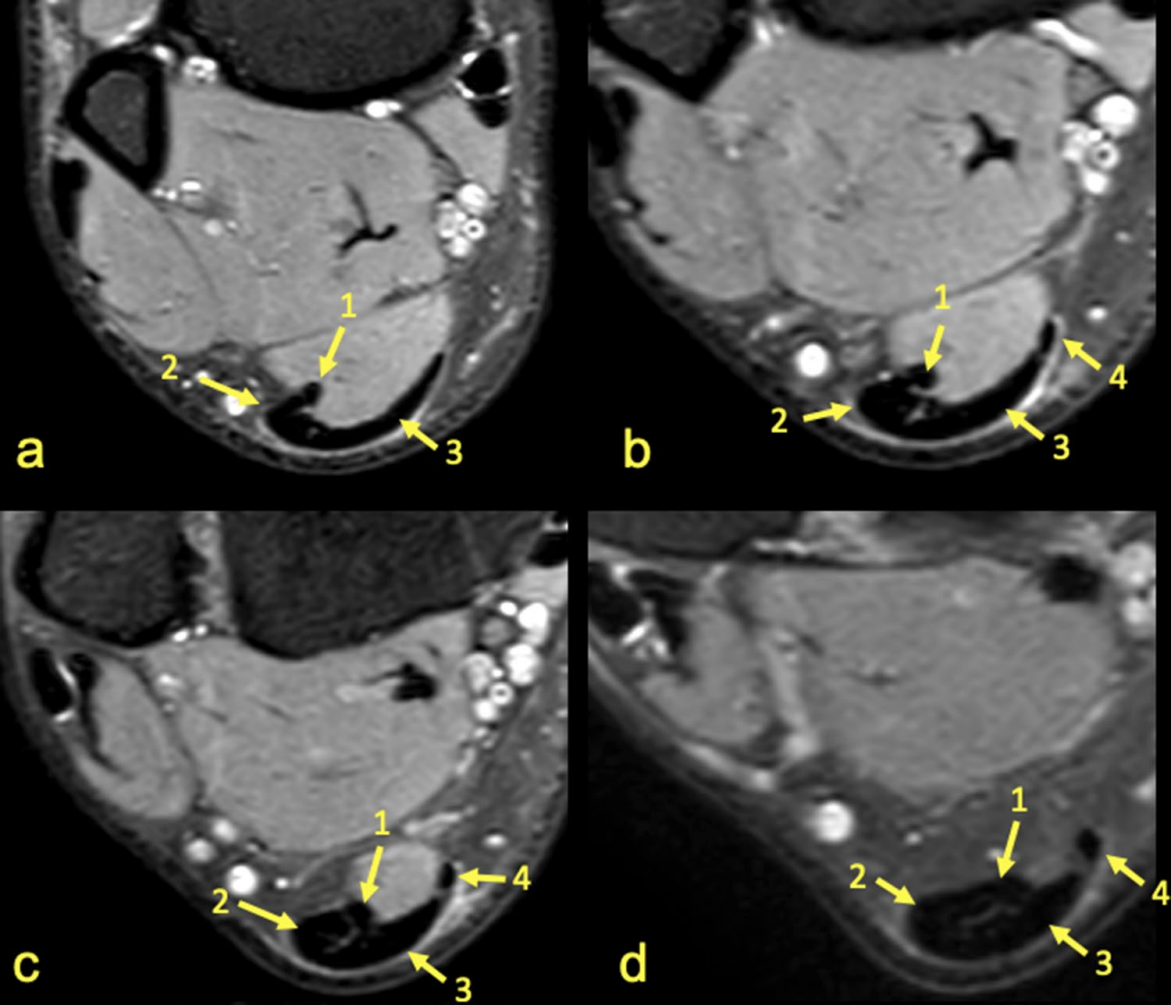
MRI right ankle, PD FS at the level of the distal tibia (**a**–**c**); (**a**)—the highest section, (**c**)—the lowest section, and the talus (**d**). A 39-year-old female 4 weeks after ankle sprain. 1—S-Sol, 2—S-LGC, 3—S-MGC, 4—the plantaris tendon (type 4).

**Figure 7:**
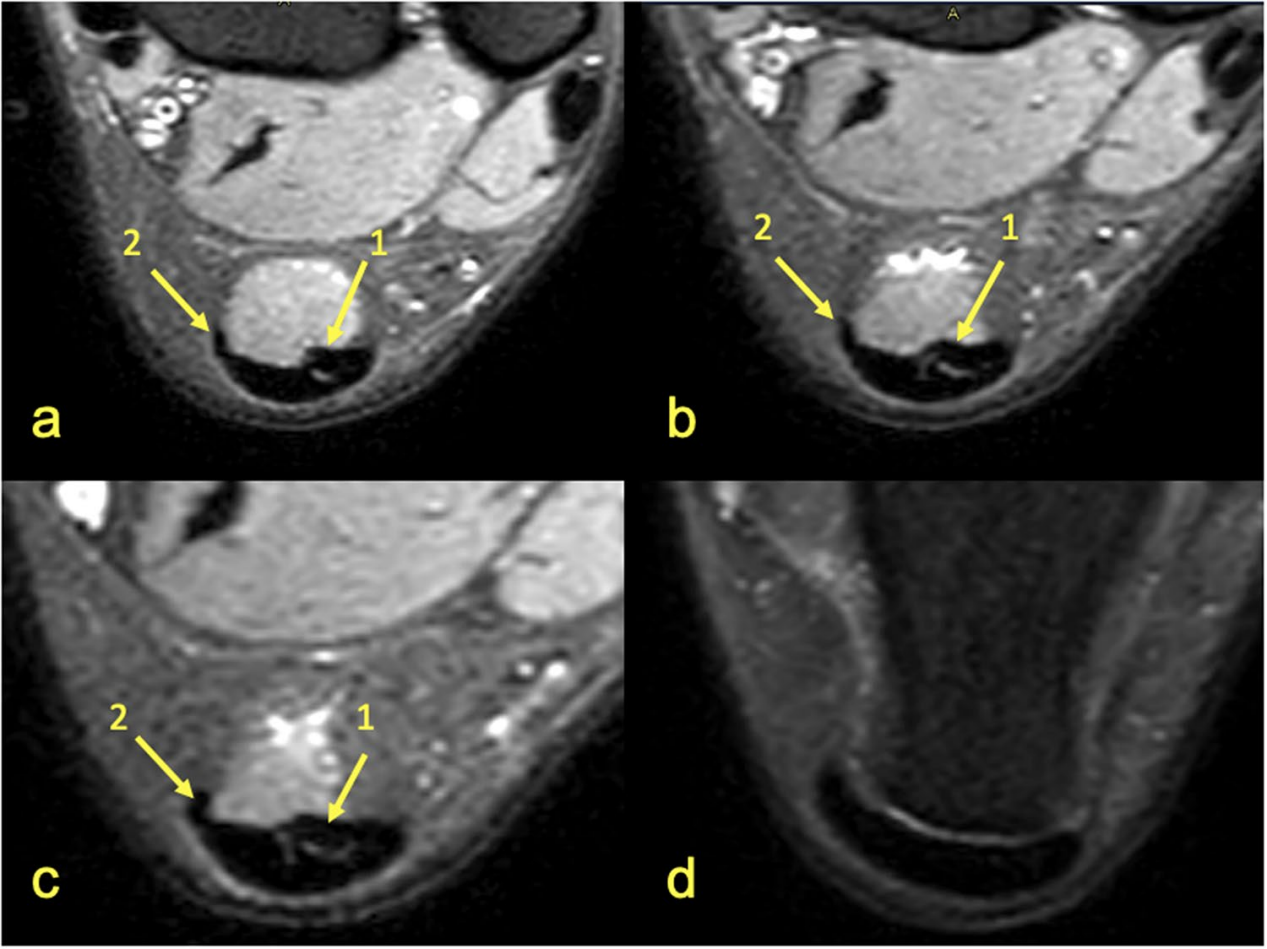
MRI left ankle, PD FS at the level of the distal tibia (**a**) is a slightly higher section than (**b**), the talus (**c**) and the calcaneus (**d**). A 37-year-old male with clinical suspicion of anterior conflict. The central tendon of the soleus muscle was absent. 1—S-LGC, 2—the plantaris tendon (type 4).

**Figure 8:**
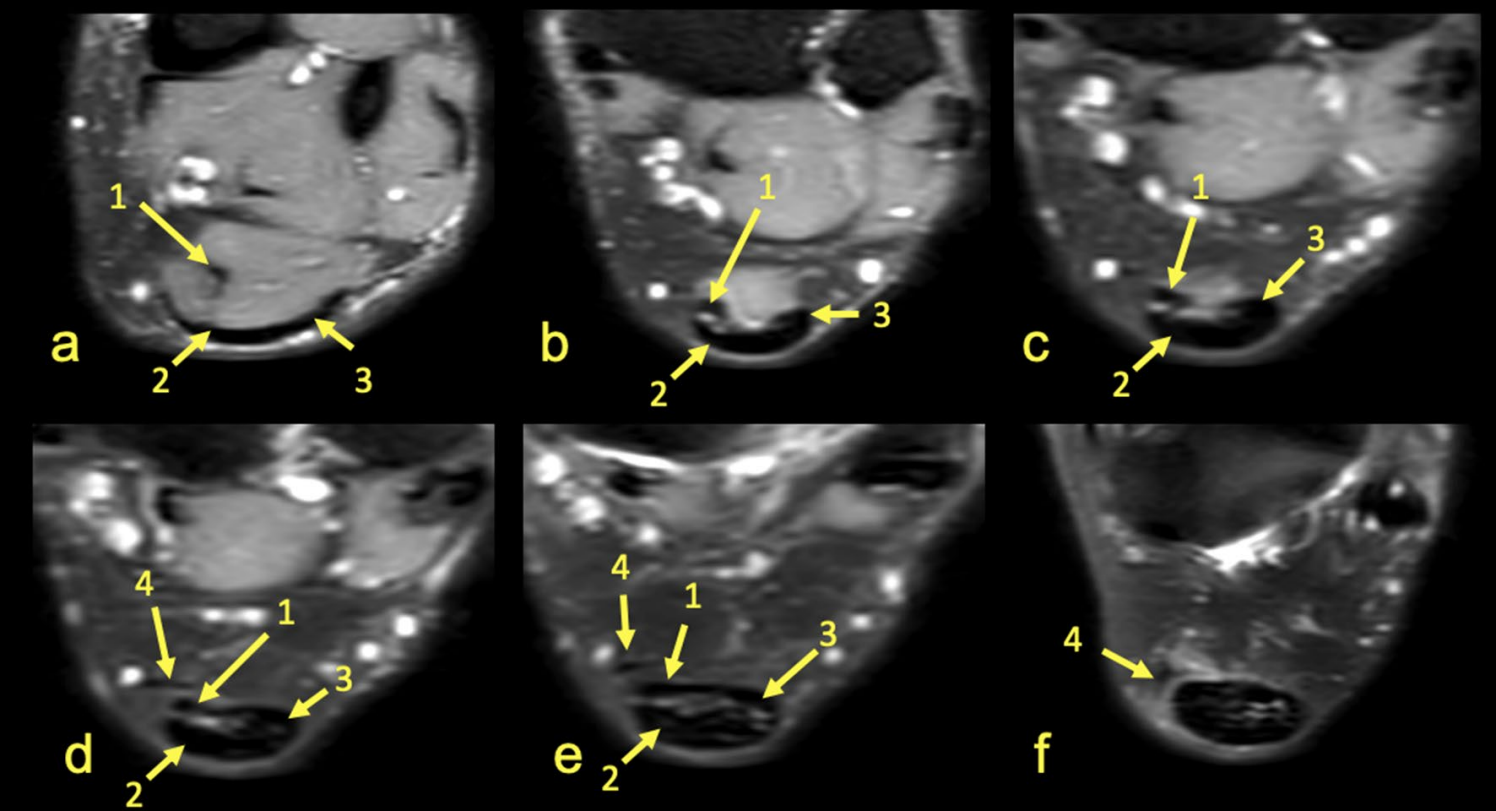
MRI left ankle, PD FS at the level of the distal tibia (**a**–**e**), the talus (**f**); (**a**)—the highest section, (**f**)—the lowest section. 1—T-Sol, 2—S-MGC, 3—S-LGC, 4—the plantaris tendon (type 3).

The anterior outline of the Achilles tendon is always comprised by two subtendons, one from the lateral head of the gastrocnemius and the other from the soleus muscle, with dominance of the first one in n = 114 cases (57%) (Figs. 2, 3, 4, 5, 7, 8). Less often, the anterior outline is comprised mainly by the subtendon originating from the soleus muscle (Fig. 6). The posterior outline of the Achilles tendon is composed of the S-MGC (Figs. 6, 8).

The posterior outline of the Achilles tendon is composed by the S-MGC, the medial outline by the S-Sol while the anterior by the S-LGC and S-Sol. The lateral outline of the Achilles tendon is formed by the S-LGC and S-MGC.

**Figure 9:**
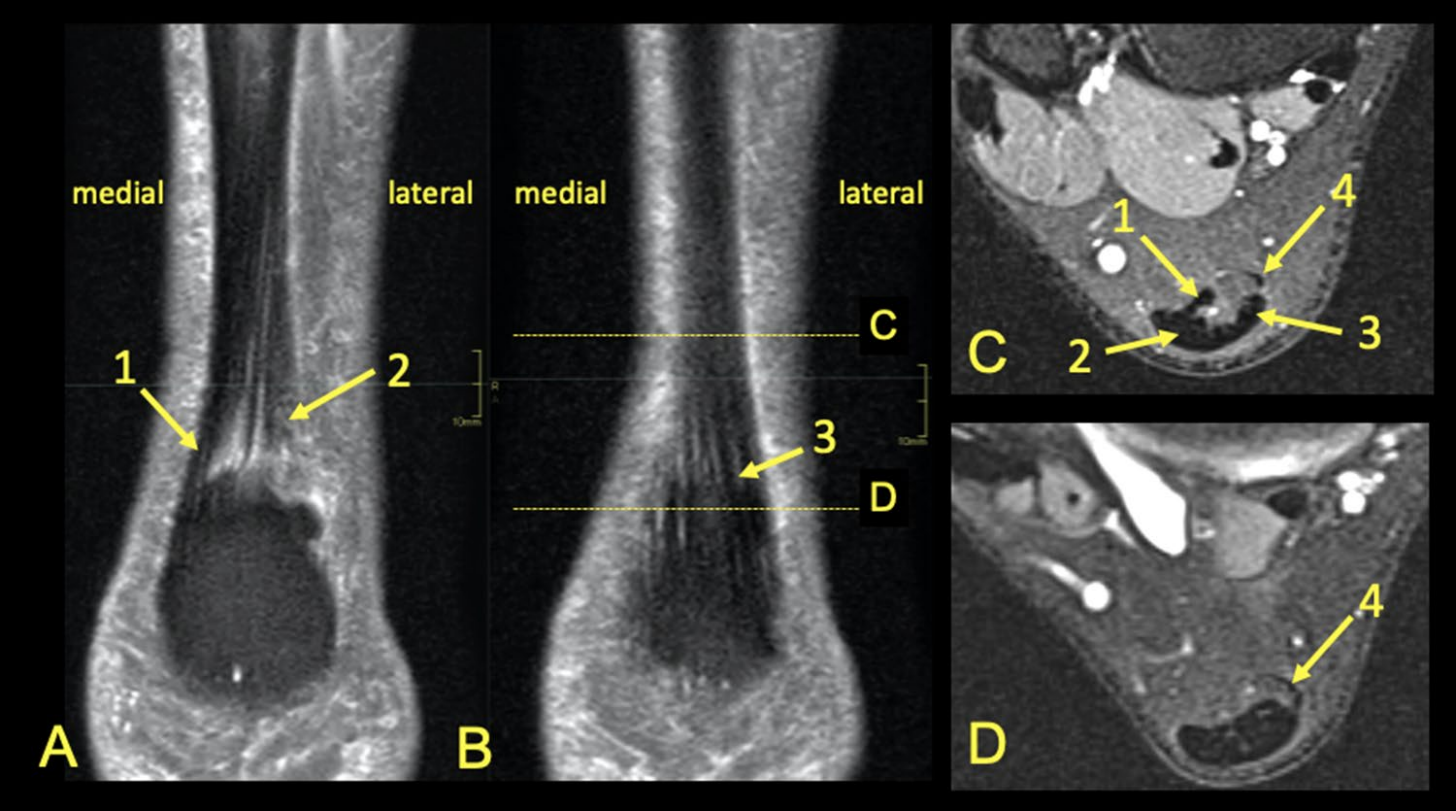
MRI right Achilles tendon, PD FS. The words medial and lateral are given for better orientation. (**A**,**B**) coronal picture of the right Achilles tendon. (**C**,**D**) transverse section at level shown on (**B**). 1—fibers from the soleus muscle, 2—fibers from the lateral head of the gastrocnemius muscle, 3—fibers from the medial head of the gastrocnemius muscle, 4—the plantaris tendon.

**Figure 10:**
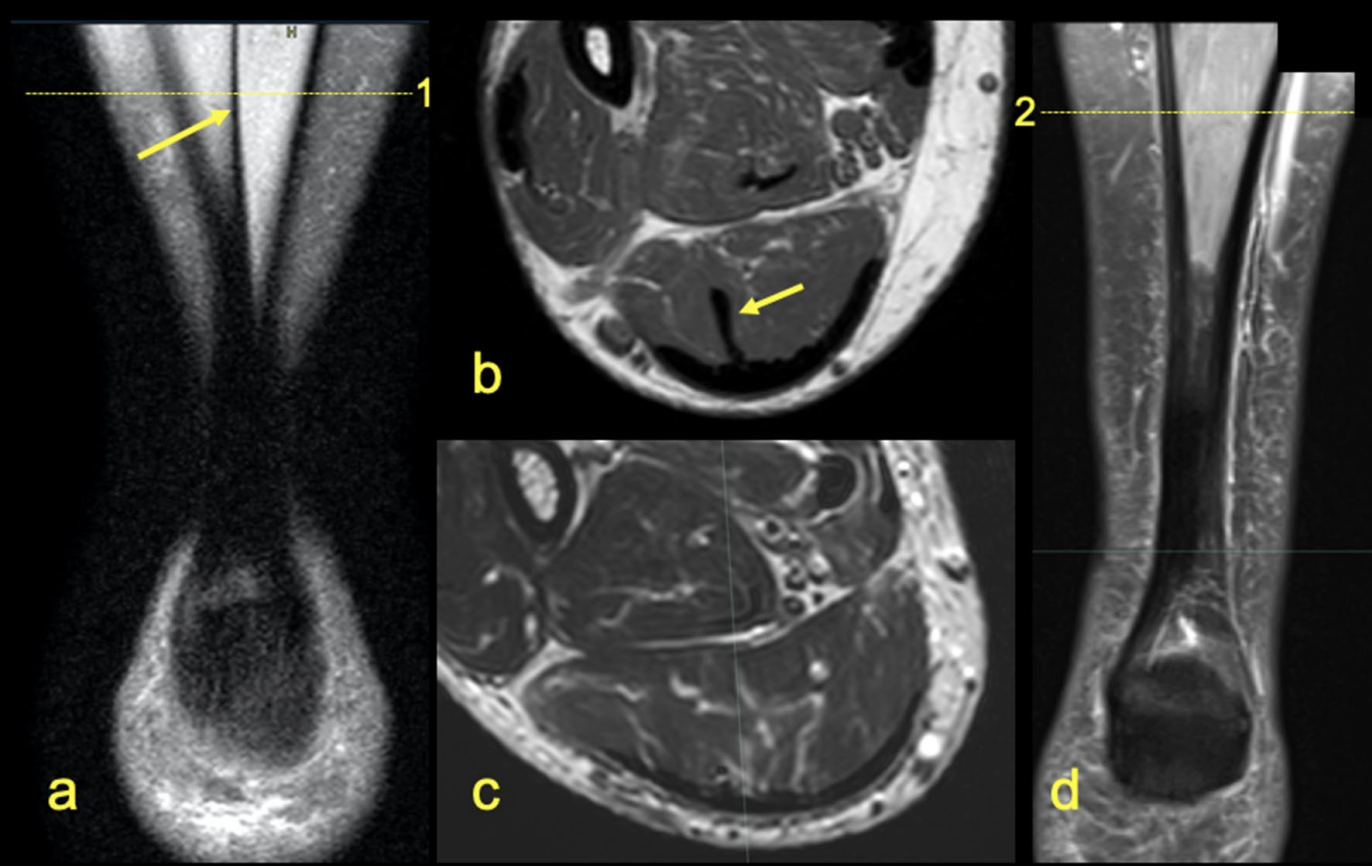
Two patients. One presented on (**a**,**b**) with the central tendon of the soleus muscle marked by an arrow. The other on (**c**,**d**) shows the absence of the central tendon of the soleus muscle.

The presence of the T-Sol was found in n = 170 cases (85%) (Figs. 9, 10), however, only a weak statistically significant correlation between the presence of the T-Sol (Fig. 9) and the possibility of identifying the S-Sol within the Achilles tendon was demonstrated (r = 0.15, *P* > 0.05). No statistically significant correlation was found between the side or gender of the patient and the possibility of identifying the subtendons within the Achilles tendon.

### Connection of the plantaris tendon to the Achilles tendon

**Figure 11:**
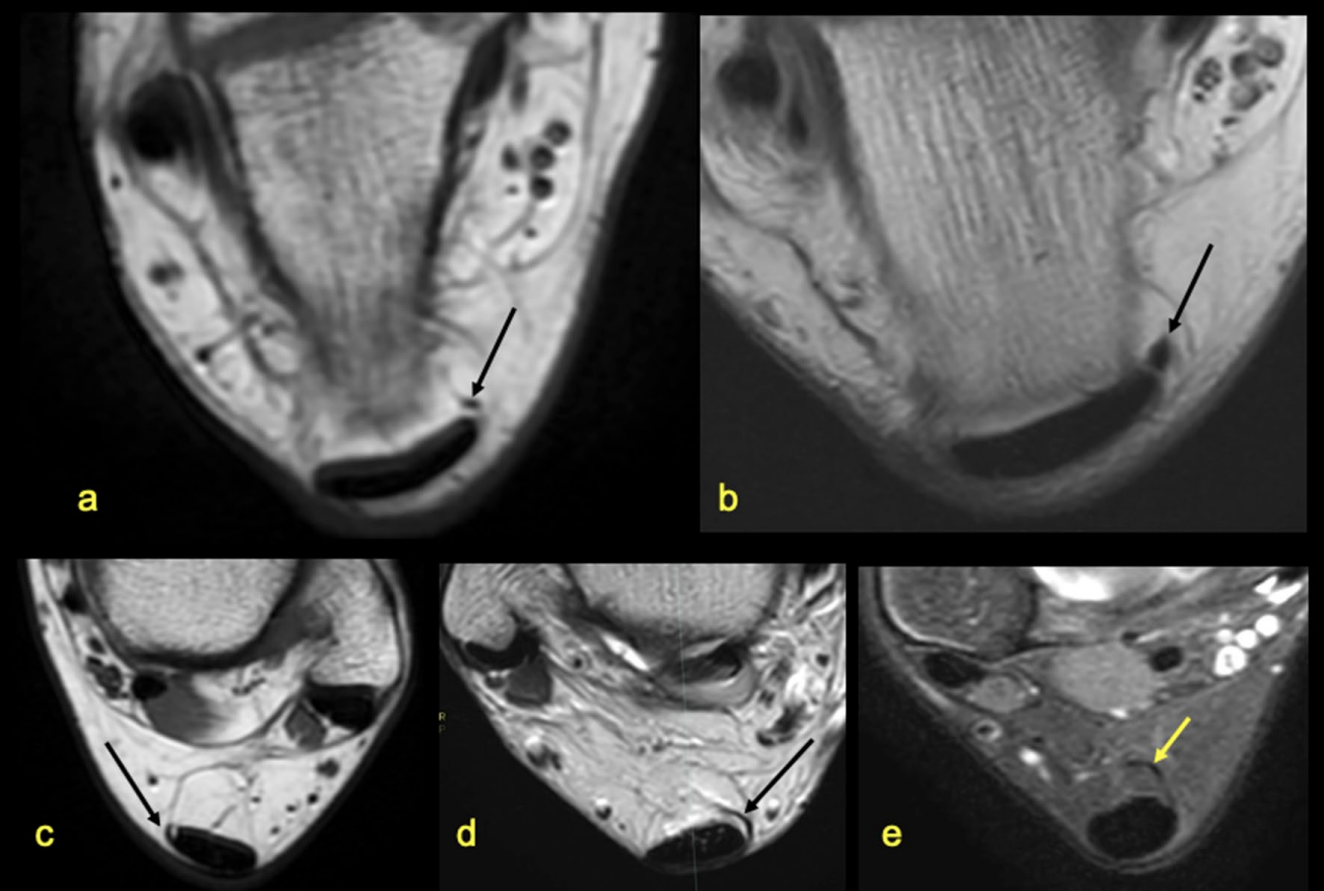
Five variations of the plantaris tendon. The most common seen variations (**a**) (type 3) and (**b**) (type 4). The less often seen variations (**c**) (type 1), (**d**) (type 2) and (**e**) (type 5).

We identified five variations of the plantaris tendon (Table 1, Figs. 4, 5, 6, 7, 8, 11). The direct connection of the plantaris tendon to the Achilles tendon was seen in type 2 and 4, n = 89 cases (42.5%). Weak statistically significant positive correlation is visible between the possibility of visualizing the subtendon from the lateral head of the gastrocnemius muscle and types 3 and 4 of the plantaris tendon (Table 3). In contrast, a negative weak correlation occurs between type 5 or the absence of the plantar tendon.

**Table 3:** R Pearson value for the correlation of identification of the subtendons of the Achilles tendon and the absence or variations of the plantaris tendon (Fig. 11).

	absent	Type 2	Type 3	Type 4	Type 5
S-MGC	0.08	− 0.04	0.06	0.02	− 0.07
S-LGC	− 0.24	0.04	0.21	0.21	− 0.29
S-Sol	0.08	− 0.05	0.04	− 0.09	0.10

*P* < 0.05. Type 1 of the plantaris tendon is not included because of the small number of cases.

The final consensus of the possibility of identification of the subtendons of the Achilles tendon, the central tendon of the soleus muscle and variation of the plantaris is presented in Table 1.

### Rotation of the subtendons in the Achilles tendon

The angles between the long axis of the tibia and long axes of each subtendon were measured on axial and coronal sections (Figs. 1, 2, 3, 4, 5, 6, 7, 8, Table 4). The Achilles tendon was visible on 2 (n = 49 cases) or 3 (n = 151) coronal slides. The mean angles of the long axis of the subtendons are given in Table 4. R Pearson values near to zero were noted in correlation with the subtendon’s angles in correlation to gender and side, consequently no explicit differences were noted (Table 4).

**Table 4:** Values of angle of the Achilles tendon subtendons in relation to the long axis of the tibia.

Side and gender	Value	S-MGC	S-LGC	S-Sol
Right side	Angle mean	12.7°	9.9°	3.9°
	Angle min	7.2°	8.1°	2.2°
	Angle max	18.7°	10.6°	12.4°
	R Pearson	0.04	0.01	− 0.03
Left side	Angle mean	13.3°	10.1°	4.3°
	Angle min	3.9°	6.3°	3.1°
	Angle max	17.3°	14.2°	11.3°
	R Pearson	− 0.04	− 0.01	0.03
Right and left	Angle mean	11.1°	9.1°	6.6°
Female	R Pearson	0.06	− 0.03	− 0.02
Male	R Pearson	− 0.06	0.03	0.02

Correlation of side and gender with the angles of the subtendons, *P* < 0.05.

## Discussion

The distribution of the subtendons of the midportion the Achilles tendon found in our study is the following the S-Sol is located antero-medially, the S-LGC anteriorly while the S-MGC posteriorly. The posterior outline of the Achilles tendon is formed by the S-MGC, the medial outline by the S-Sol, the anterior by the S-LGC and S-Sol. The lateral outline is formed by the S-LGC and S-MGC. The internal structure and position of the subtendons of the normal Achilles tendon has not yet been studied on MRI in larger material before. Most of the previous studies assessing the internal structure of the Achilles tenon were conducted on cadavers^4,5,16–20^ and very few in ultrasound^8^. We think that our study is necessary due to the fact that MRI is a modern method of imaging the musculoskeletal and an excellent tool for assessing the Achilles tendon anatomy^4,5,21^. We noticed that the possibility of identifying the subtendons on MRI is not as clear as during an anatomical dissection and classical anatomy of the Achilles tendon is significantly different from then radiological anatomy. Identification of the subtendons of the Achilles tendon on MRI depends on the presence of narrow high-signal septations between the subtendons^21^ and the ability to identify is partly dependent on the reviewer. No large-scale MRI studies such as this have been previously reported. We studied the Achilles tendon between the lower outline of the soleus muscle belly to the level of the calcaneal tuber, which includes the zone where tendinopathy and ruptures most often occur^2,8,22^. Active tracking of the subtendons from the myotendinous junction, distal to the calcaneus, showed the same twisted structure of the Achilles tendon as revealed previously^5,10,17,18,20,23^. Most often it was possible to determinate the subtendon originating from the lateral head of the gastrocnemius muscle. The revealed subtendon’s location described in previous studies^4,17,20^ was consistent with our MRI findings. The localization of the subtendons revealed in our study is the following: the subtendon from the lateral head of the gastrocnemius muscle is located in the anterior part of the Achilles tendon, the subtendon from the soleus muscle is located medially to this, while the posterior part of the Achilles tendon is comprised of the subtendon from the medial head of the gastrocnemius muscle. We found intermediate to good interobserver agreement values (k = 0.58–0.75). We do not think that different MRI sequences or other cross-section or section thicknesses would improve agreement but that would presumably improve the assessment of the angles.

In contrast to anatomical studies^4,17–20^ we were not able to distinguish the subtendons at the level of bone attachment because the tendon structure is quite compact and the position of the subtendons at the insertion can be only presumed on MRI^16,17^. The tight packing of the fascicles of the Achilles tendon at the insertion may explain why the subtendons could not be identified. The Achilles tendon shows symptoms of diseases affecting surrounding structures, e.g. retrocalcaneal bursitis or Haglund deformity. This area is also quite sensitive to partial injuries causing isolated laxity of the tendon^22^. The largely uniform structure of the Achilles tendon is only apparent because the nonuniform function of the Achilles tendon occurs during movement and exercise^23^ and internal tendon tears are longitudinal in direction^24^.

In our study, we revealed that the anterior outline of the Achilles tendon is formed laterally by the subtendon originating from the lateral head of the gastrocnemius muscle while the subtendon of the soleus muscle is located anteriorly and medially. This structure is similar to the results of previous anatomical examinations^4,17,18,20^. Usually, dominance of the subtendon from the lateral gastrocnemius muscle was observed which corresponds to type II according to Edama^17,18^. In no case was the subtendon from the medial head of the gastrocnemius muscle divided into parts as was previously suggested^4^. This difference may be due to the fact that the MRI examination showed poor fiber rotation within the subtendon, whereas the configuration of superficial fibers is clearly visible on gross anatomy specimens. This is probably also the reason for the inability to compare with the results of previous anatomical studies^19–21^. Several anatomical studies have shown different degrees of the Achilles tendon twist structure, but there are no studies assessing the tendon twist on MRI. Previous anatomical studies assessing the rotation of the tendon fibers give very divergent results from 5 to 90 degrees^17–20^. On MRI, it is not possible to distinguish between fibers in the subtendons, but this is not difficult during anatomical dissection. The appropriate method of angle measuring should consider the three-dimensional nature of the Achilles tendon^19^. The angles measured in our study were estimated based on the axial sections and two or three coronal sections, which is a projection of the twisted tendon surface to a flat view plane which is a limitation of this study. Our results also differ from most anatomical works because we measured the angle of the subtendon’s axis, not the angle of the superficial fibers or the fiber rotation as in most previous anatomical studies. On the other hand, counting only superficial fibers orientation may lead to misinterpretation of the tendon rotation. The results of our study are comparable only with the work published Prosenz et al.^21^, where the internal structure of the subtendons was examined. A solution could be the use of ultra-thin MRI sections of the Achilles tendon, which is not routinely used clinically.

In occasional anatomical studies, variability in the degree of tendon torsion was observed, separating the types of torsion variability^19,21^. In our material we did not find cases where only one subtendon formed the anterior outline of the Achilles tendon, which was revealed by some authors^17^. Only a variable size ratio between the subtendons originating from the soleus and the lateral head of the gastrocnemius muscles was observed.

Previous studies^17^ have not shown statistically significant differences in the twisted structure between the sexes and sites examined, however, comparison with our work on larger material and another method is significantly limited. In our study, statistical analysis showed virtually no correlation between torsion of tendon and gender or side.

We decided to assess the location of the plantaris tendon in relation to the Achilles tendon due to its potential role in the medial Achilles tendinopathy^8,15^. The connection of the plantar tendon with the Achilles tendon was seen relatively often (type 2 and 4) most often as a common insertion to the calcaneal tuber or as a contribution to the medial outline of the Achilles tendon. Considering the methodology of our study, we decided to use the plantaris tendon classification according to Olewnik et al.^15^, which is a good compromise between the classification according to Cummins and Anson^25^ and one according to Sterkenburg et al.^26^. The reason is that MRI has a lower sensitivity than dissection for the visualization of small tendon connections which is described in some rare variants^15,26^.

There was no correlation between the possibility of subtendon identification and the side, gender, presence of the central tendon of the soleus muscle or the anatomical variant of the plantaris tendon. According to our knowledge, no studies of similar relationships were carried out either on anatomical specimens or MRI.

The limitations of our study were: its retrospective character and no anatomical-surgical correlation.

Classical and radiological anatomy of the Achilles tendon are different. Identification of the individual subtendons in a healthy Achilles tendon on MRI is possible above the calcaneus, although it is challenging. Most often only the subtendon from the lateral head of the gastrocnemius muscle can be identified, which, together with the subtendon from the soleus muscle, forms the anterior outline of the Achilles tendon. The posterior outline of the Achilles tendon is formed by the subtendon from the medial head of the gastrocnemius muscle. In most cases, the plantaris tendon has a direct connection to the medial part of the Achilles tendon. The application of the results of our study allows a more accurate association of the subtendons with individual parts of the triceps surae muscle. This is very valuable knowledge in prevention the development of overuse, tendinopathy and Achilles tendon ruptures.

## Methods

We conducted the retrospective study of 200 ankle MRI of athletes (112 males and 88 females; the average age 29 (range 16–47) years; 101 right ankles and 99 left ones) without history of trauma or obvious abnormality in relation to the Achilles tendon. Only patients without tendinopathy of the Achilles tendon and with complete clinical data were included.

MRI examinations were performed at our institution over six months (between November 2018 and April 2019), about 2–4 weeks after the ankle injury (mean duration 2.8 weeks) for clinical purposes. All examinations were performed using a 3.0-T MRI scanner. The systematic re-evaluation was conducted by two independent observers. We used Cohen’s Kappa coefficient to determine inter-observer agreement. A coefficient was calculated for each subtendon, the central soleus tendon and each type of soleus. The kappa coefficient ranges from − 1 to + 1, agreements may be qualified as: excellent (> 0.81), good (0.61–0.8), intermediate (0.21–0.60), or poor (< 0.21)^27^. Final determination of the subtendons was made by consensus.

Due to the known internal structure of the Achilles tendon^4,20^, T2-weighted or PD (proton density) images on the frontal and transverse planes were used. On the coronal plane, it was assessed whether the rotation of the subtendons could be identified, then the position of the soleus central tendon was identified.

The angles between the long axis of the tibia and long axes of each subtendon were measured. Active tracking enables the determination of the geometric center of each subtendon at the highest possible cross-section (called X1). Then each subtendon was tracked on subsequent lower axial sections for the S-MGC and S-LGC and on coronal sections for T-Sol. Afterwards, a point X1 of the subtendon was connected with its point X2 to define the axis of the subtendon.

On the transverse plane, we assessed whether the subtendons described in the literature could be distinguished on MRI and whether their location was consistent with the previously described Achilles tendon structure. At a later stage, the relationship of the plantaris tendon to the Achilles tendon was determined, using the plantaris tendon classification according Olewnik et al.^15^.

The methods were carried out in accordance with the relevant guidelines and regulations, and informed consent was obtained from all participants, and for participants under 18, from a parent and/or legal guardian.

### Ethics approval

Ethical approval was waived by the Institutional Ethics Committee at Medical University of Warsaw, Poland in view of the retrospective nature of the study and that all the procedures being performed were part of routine care. The Institutional Ethics Committee at Medical University of Warsaw, Poland was informed about the ongoing retrospective study and the Committee stated there was no need for its approval (AKBE/258/2019).

## Data Availability

The datasets generated during and/or analysed during the current study are available from the corresponding author on reasonable request.

## References

[1] Ganestam A, Kallemose T, Troelsen A, Barfod KW (2016). Increasing incidence of acute Achilles tendon rupture and a noticeable decline in surgical treatment from 1994 to 2013. A nationwide registry study of 33,160 patients. Knee Surg. Sports Traumatol. Arthrosc..

[2] Karlsson, J., Westin, O., Carmont, M. & Nilsson-Helander, K. in *Sports Injuries of the Foot and Ankle* Ch. Chapter 33, 369–376 (2019).

[3] Lantto I, Heikkinen J, Flinkkila T, Ohtonen P, Leppilahti J (2015). Epidemiology of Achilles tendon ruptures: Increasing incidence over a 33-year period. Scand. J. Med. Sci. Sports.

[4] Szaro P, Witkowski G, Smigielski R, Krajewski P, Ciszek B (2009). Fascicles of the adult human Achilles tendon—An anatomical study. Ann. Anat..

[5] Winnicki K, Ochala-Klos A, Rutowicz B, Pekala PA, Tomaszewski KA (2020). Functional anatomy, histology and biomechanics of the human Achilles tendon—A comprehensive review. Ann. Anat..

[6] Balius R (2013). The soleus muscle: MRI, anatomic and histologic findings in cadavers with clinical correlation of strain injury distribution. Skelet. Radiol..

[7] Bolsterlee B (2018). Three-dimensional architecture of the whole human soleus muscle in vivo. PeerJ.

[8] Counsel P, Comin J, Davenport M, Connell D (2015). Pattern of Fascicular involvement in midportion Achilles tendinopathy at ultrasound. Sports Health.

[9] Hatzantonis C, Agur A, Naraghi A, Gautier S, McKee N (2011). Dissecting the accessory soleus muscle: A literature review, cadaveric study, and imaging study. Clin. Anat..

[10] Pierre-Jerome C, Moncayo V, Terk MR (2010). MRI of the Achilles tendon: A comprehensive review of the anatomy, biomechanics, and imaging of overuse tendinopathies. Acta Radiol..

[11] Garras DN, Raikin SM, Bhat SB, Taweel N, Karanjia H (2012). MRI is unnecessary for diagnosing acute Achilles tendon ruptures: Clinical diagnostic criteria. Clin. Orthop. Relat. Res..

[12] Couppe C, Svensson RB, Josefsen CO, Kjeldgaard E, Magnusson SP (2020). Ultrasound speckle tracking of Achilles tendon in individuals with unilateral tendinopathy: A pilot study. Eur. J. Appl. Physiol..

[13] Han M, Larson PE, Liu J, Krug R (2014). Depiction of achilles tendon microstructure in vivo using high-resolution 3-dimensional ultrashort echo-time magnetic resonance imaging at 7 T. Investig. Radiol..

[14] Robson MD, Benjamin M, Gishen P, Bydder GM (2004). Magnetic resonance imaging of the Achilles tendon using ultrashort TE (UTE) pulse sequences. Clin. Radiol..

[15] Olewnik L, Wysiadecki G, Polguj M, Topol M (2017). Anatomic study suggests that the morphology of the plantaris tendon may be related to *Achilles tendonitis*. Surg. Radiol. Anat..

[16] Ballal MS, Walker CR, Molloy AP (2014). The anatomical footprint of the Achilles tendon: A cadaveric study. Bone Joint J..

[17] Edama M (2015). The twisted structure of the human Achilles tendon. Scand. J. Med. Sci. Sports.

[18] Edama M (2016). Structure of the Achilles tendon at the insertion on the calcaneal tuberosity. J. Anat..

[19] Pekala PA (2017). The twisted structure of the Achilles tendon unraveled: A detailed quantitative and qualitative anatomical investigation. Scand. J. Med. Sci. Sports.

[20] van Gils CC, Steed RH, Page JC (1996). Torsion of the human achilles tendon. J. Foot Ankle Surg..

[21] Prosenz J, Rath C, Hadrovic-Avdic M, Hirtler L (2018). The twist of the achilles tendon—Associations of torsions in the lower extremity. Clin. Anat..

[22] Mahan J (2020). Achilles tendon complex: The anatomy of its insertional footprint on the calcaneus and clinical implications. J. Orthop..

[23] Handsfield GG (2017). A 3D model of the Achilles tendon to determine the mechanisms underlying nonuniform tendon displacements. J. Biomech..

[24] Chan O (2017). Intratendinous tears of the Achilles tendon—A new pathology? Analysis of a large 4-year cohort. Muscles Ligam. Tendons J..

[25] Cummins Ej Fau-Anson BJ, Anson BJ (1946). The structure of the calcaneal tendon (of Achilles) in relation to orthopedic surgery, with additional observations on the plantaris muscle. Surg. Gynecol. Obstet..

[26] van Sterkenburg MN, Kerkhoffs GM, Kleipool RP, van Dijk CN (2011). The plantaris tendon and a potential role in mid-portion Achilles tendinopathy: An observational anatomical study. J. Anat..

[27] Landis JR, Koch GG (1977). The measurement of observer agreement for categorical data. Biometrics.

